# Comprehensive Uncertainty Quantification and Sensitivity Analysis for Cardiac Action Potential Models

**DOI:** 10.3389/fphys.2019.00721

**Published:** 2019-06-26

**Authors:** Pras Pathmanathan, Jonathan M. Cordeiro, Richard A. Gray

**Affiliations:** ^1^Center for Devices and Radiological Health, U.S. Food and Drug Administration, Silver Spring, MD, United States; ^2^Masonic Medical Research Institute, Utica, NY, United States

**Keywords:** simulation, electrophysiology, credibility, robustness, canine

## Abstract

Recent efforts to ensure the reliability of computational model-based predictions in healthcare, such as the ASME V&V40 Standard, emphasize the importance of uncertainty quantification (UQ) and sensitivity analysis (SA) when evaluating computational models. UQ involves empirically determining the uncertainty in model inputs—typically resulting from natural variability or measurement error—and then calculating the resultant uncertainty in model outputs. SA involves calculating how uncertainty in model outputs can be apportioned to input uncertainty. Rigorous comprehensive UQ/SA provides confidence that model-based decisions are robust to underlying uncertainties. However, comprehensive UQ/SA is not currently feasible for whole heart models, due to numerous factors including model complexity and difficulty in measuring variability in the many parameters. Here, we present a significant step to developing a framework to overcome these limitations. We: (i) developed a novel action potential (AP) model of moderate complexity (six currents, seven variables, 36 parameters); (ii) prescribed input variability for all parameters (not empirically derived); (iii) used a single “hyper-parameter” to study increasing levels of parameter uncertainty; (iv) performed UQ and SA for a range of model-derived quantities with physiological relevance; and (v) present quantitative and qualitative ways to analyze different behaviors that occur under parameter uncertainty, including “model failure”. This is the first time uncertainty in every parameter (including conductances, steady-state parameters, and time constant parameters) of every ionic current in a cardiac model has been studied. This approach allowed us to demonstrate that, for this model, the simulated AP is fully robust to low levels of parameter uncertainty — to our knowledge the first time this has been shown of any cardiac model. A range of dynamics was observed at larger parameter uncertainty (e.g., oscillatory dynamics); analysis revealed that five parameters were highly influential in these dynamics. Overall, we demonstrate feasibility of performing comprehensive UQ/SA for cardiac cell models and demonstrate how to assess robustness and overcome model failure when performing cardiac UQ analyses. The approach presented here represents an important and significant step toward the development of model-based clinical tools which are demonstrably robust to all underlying uncertainties and therefore more reliable in safety-critical decision-making.

## 1. Introduction

Computational modeling and simulation (M&S) is a powerful tool for medical product design optimization, safety evaluation, clinical trial reduction, and enabling precision medicine (Viceconti et al., 2016; Faris and Shuren, 2017; Morrison et al., 2018). Lately, several initiatives have aimed to advance biomedical M&S by developing and promoting best practices and methods for rigorously assessing the credibility–that is, the trustworthiness–of computational models. These include: the ASME V&V40 Standard (ASME V&V 40, 2018), a new consensus Standard developed by the medical device community which outlines a framework for credibility assessment for models with medical device applications; reports advocating for formalized methods and education into credibility assessment (National Research Council, 2012); and research related to model credibility across a variety of biomedical fields (Hariharan et al., 2015; Hicks et al., 2015; Collis et al., 2017; Pathmanathan et al., 2017; Patterson and Whelan, 2017; Mulugeta et al., 2018). Demonstrating or evaluating model credibility is challenging for models with biomedical applications, but it can be especially difficult for physiological models—models that simulate physiological function, as opposed to traditional physics-based models such as solid mechanics or fluid dynamics models. One important specialty within physiological modeling is cardiac modeling. Cardiac electrophysiology (EP) models have been an essential tool for basic cardiac research for over half a century and recently have transitioned into regulatory and clinical applications (Niederer et al., 2018). In particular, the Comprehensive *in vitro* Pro-arrhythmia Assay (CiPA) program proposes to replace the long QT study based paradigm for assessing cardiotoxicity of novel compounds with a series of *in vitro* and *in silico* tests, one of which uses simulation of drug effects on the action potential using a cardiac cellular model (Li et al., 2018; Strauss et al., 2018). Another notable recent breakthrough is research demonstrating the clinical predictive capability of personalized whole-heart models in patient stratification (Arevalo et al., 2016) and other clinical cardiology applications (Ashikaga et al., 2013). Clinical trials are currently underway evaluating the ability of personalized heart models to guide ablation therapy^1^. In parallel, there has been growing interest and research into cardiac model credibility (Niederer et al., 2011; Pathmanathan and Gray, 2013, 2018; Krishnamoorthi et al., 2014; Mirams et al., 2016).

One important aspect to model credibility assessment is the study of *uncertainty*. Parameters in physiological models are often uncertain due to either measurement uncertainty and/or natural physiological variability. Uncertainty quantification (UQ) and sensitivity analysis (SA) are two related tasks for studying uncertainty. UQ is the process of determining the uncertainty in model inputs, and then estimating the resultant uncertainty in model outputs. SA appears to have different interpretations to different communities (discussed in more detail below), but fundamentally is the study of which inputs most affect a model output. Overall, UQ and SA test the robustness of model predictions, for example revealing if predictions are unacceptably wide-ranging when input uncertainty is accounted for. UQ replaces a traditional *deterministic* approach to modeling where inputs and outputs take fixed values, with a *probabilistic* approach in which uncertainty in inputs and outputs are known, thereby providing a deeper understanding of system behavior. For example, UQ in weather forecasting leads to probabilities of weather events (e.g., probability of rain) being presented to the public, which is much more useful that simple predictions (e.g., “will rain”/“will not rain”). Therefore, the ASME V&V40 Standard asks the analyst to consider whether UQ and SA was performed and how comprehensive the analysis was (ASME V&V 40, 2018). Similarly, there is an increased awareness of the need for UQ by funding agencies, for example a recent call for physiological models to support the development of the next generation of neuromodulation devices^2^ states one goal as “build uncertainty quantification into composite model outputs by propagating uncertainties from *all* component model parameters” [emphasis added]. However, there are numerous challenges to performing UQ and SA for physiological models, and UQ in particular is not yet well-established. In this paper, we will use a novel cardiac cell model and study the impact of interacting uncertainty in all model parameters, on the simulated action potential. Before explicitly stating our aims, we introduce UQ and SA in more detail, and provide a motivating discussion of what ‘comprehensive’ UQ for whole heart modeling would require.

### 1.1. Uncertainty Quantification and Sensitivity Analysis

There are two stages to UQ, which we will refer to as *uncertainty characterization* (UC) and *uncertainty propagation* (UP). Uncertainty characterization is the quantification of the uncertainty in model *inputs*. Here, “inputs” is a broad term for any quantity in the model whose *value is based on real-world data*. This includes model parameters, boundary and initial conditions, loading conditions, underlying geometry and material properties. The most common reasons for uncertainty in a model input are measurement uncertainty (e.g., the inability to measure a quantity exactly) and natural variability (e.g., variability in a physiological quantity across individuals in a population). The aim of UC is to determine probability distributions describing each of the inputs. This is generally a data-driven task that can be especially difficult for complex models with large numbers of parameters, where even estimating mean values can be challenging. The second stage, uncertainty propagation, involves propagating the input uncertainty through the model to derive the resultant uncertainty in important model *outputs*. This can be a computationally-challenging task, since it typically requires large numbers of simulations to be run. For a detailed introduction to UQ see Smith (2013).

There appear to be different interpretations of sensitivity analysis by different communities, and unfortunately no consistent terminology for distinguishing between them. In one interpretation, SA uses the distributions for model inputs (identified in the UC stage as discussed above), and involves calculating how the uncertainty in the model output can be apportioned to the uncertainties in inputs (Saltelli et al., 2008). In this interpretation, SA and UP are complementary activities: first UC is performed, then UP calculates the output uncertainty and SA identifies which inputs are responsible for that output uncertainty. This is *global sensitivity analysis* (GSA), since the entire range of permissible parameter values is considered. Local sensitivity analysis (LSA), on the other hand, focuses on how model outputs are affected when parameters are perturbed from a nominal set of values. GSA using empirically-derived input distributions provides a fundamentally different measure of sensitivity compared to LSA, since it uses information derived from experiment that is not used in LSA^3^. For a detailed introduction to SA see Saltelli et al. (2008).

### 1.2. Hypothetical Example of Rigorous Comprehensive UQ for a Whole Heart Model

**Figure 1:**
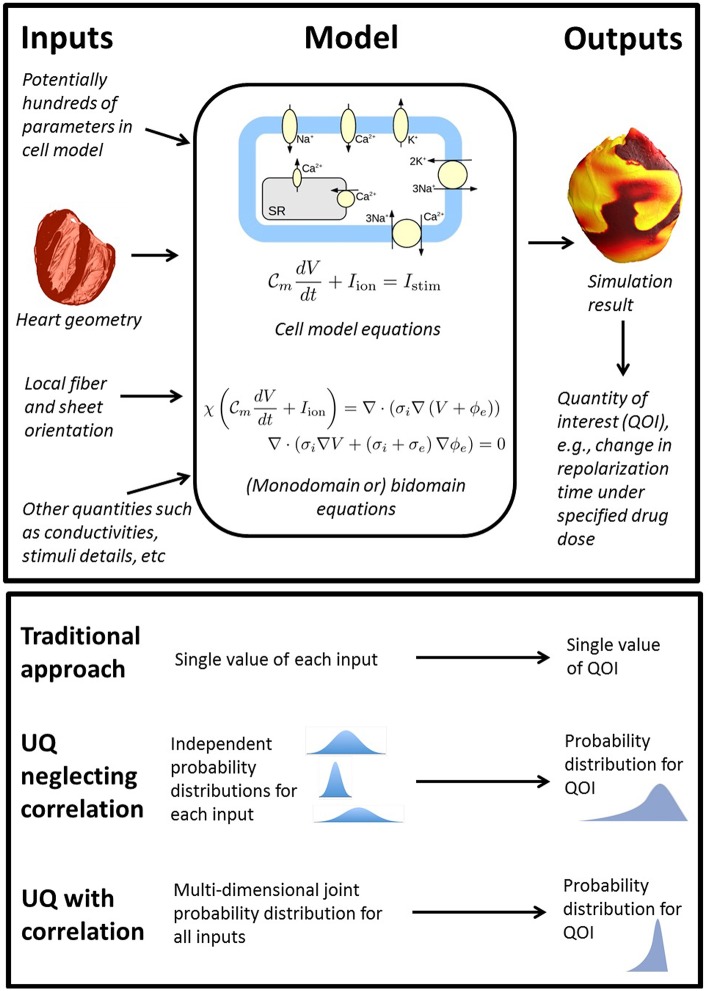
**Top**: overview of the wide range of inputs (quantities derived from experimental data) in whole heart models, all of which are uncertain to some extent, together with model equations and sample output. **Bottom**: comparison of traditional approach (no UQ), UQ neglecting potential correlations between model inputs, and UQ accounting for correlation.

To consider what rigorous fully comprehensive UQ might entail for a whole-heart EP model, first consider the components of such models as illustrated in Figure 1. Whole-heart models usually include a cellular action potential (AP) model (“cell model”), which are typically sets of ordinary differential equations (ODEs), and comprised of multiple sub-models of e.g., ion channels and sub-cellular processes. Notable human cell models proposed in recent years include the ten Tusscher model [TP06] (ten Tusscher and Panfilov, 2006) (system of 19 ODEs), the O'Hara & Rudy model [OR11] (O'Hara et al., 2011) (system of 41 ODEs) and the Grandi model (Grandi et al., 2010) (38 ODEs). These cell models are coupled to partial differential equations (PDEs) governing electrical wave propagation, usually either the monodomain or bidomain equations (Clayton et al., 2011). These are solved over a computational mesh representing the heart anatomy. To understand what a comprehensive UQ analysis might entail for a whole-heart model, consider all the inputs listed in Figure 1, using the broad definition of input as any quantity whose value is derived from real world data. All of these inputs are uncertain to some degree, and therefore a fully comprehensive UQ analysis, loosely equivalent to analyses done in other fields, would require characterization of uncertainty or variability in *all of these inputs*. This includes all the physiological parameters within the cell model (often numbering in the hundreds, for example the OR11 model has over 250 parameters), the geometry of the heart (for which there may be significant variability in shape and size across the human population), and the fiber-sheet orientation (often highly uncertain due to difficulty in measurement *in vivo*). Characterizing the uncertainty in all of these quantities is an immense experimental challenge. Moreover, a full uncertainty characterization also requires *correlations* between these quantities to be identified across the human population. For example, there is evidence for correlation of maximal conductances of *I*_Na_ and *I*_Kr_ (Milstein et al., 2012) and half-activation and half-inactivation voltages *I*_Na_ (Clerx, 2017). The inability or experimental difficulty in measuring such correlations is one of the biggest factors prohibiting comprehensive UQ with cardiac models. Correctly accounting for correlation may be key to successful UQ analysis, because, as implied in Figure 1, neglecting correlations may lead to overly wide ranging output distributions (through oversampling of non-physiological regions of parameter space).

Even if the uncertainty in all the model inputs could somehow be characterized, uncertainty propagation raises two further challenges. This first is the computational expense of running a massive number of whole heart simulations to propagate the input uncertainty through the model. Whole heart simulations are computationally expensive and generally require high performance computing resources; running millions (or more) of simulations to obtain estimates of model outputs is likely to be prohibitively expensive. Second, as the inputs are varied, it is very likely that some of the simulations will fail in some way or display a variety of behaviors (e.g., repolarization failure). Suppose this occurs during a formal UQ analysis for which the aim is to demonstrate that a model-derived conclusion (e.g., a clinical decision made using the model) is robust to the underlying uncertainties. If model failure occurs, the conclusion/decision is not robust to the input uncertainties. The second challenge is that with such complex models and such large parameter dimensions, it may be unclear how to resolve this issue when it occurs.

### 1.3. Previous Work and Study Aims

It is debatable as to whether anything similar to the above hypothetical example will ever be attained, but it is important to recognize that it is loosely equivalent to the level of UQ rigor used in other fields where modeling is used in safety critical decision making (Oberkampf and Roy, 2010). Currently, nothing that in any way resembles the above has been performed. Previous UQ/GSA research on cardiac cell models has typically involved a relatively small number of parameters *in comparison with the total* number of parameters in the model. Examples of traditional UQ analyses (i.e., empirically-derived input distributions, propagated through a model) include (Pathmanathan et al., 2015), in which UQ was performed in two parameters determining steady state *I*_Na_ inactivation, and a recent publication for the CiPA project (Chang et al., 2017), in which UQ was used to determine the impact of uncertainty in drug binding and drug ionic current block on drug risk stratification. While this provided important insight on the robustness of CiPA model predictions, it only considered uncertainty in drug related parameters, not uncertainty in the parameters of the cell model. Various alternative modeling frameworks have been devised for handling uncertainty, especially physiological variability. The population of models approach (Britton et al., 2013; Muszkiewicz et al., 2016; Passini et al., 2017) (essentially) derives distributions for certain parameters by calibration to AP recordings using acceptance/rejection criteria, and then performs UP by running simulations using the derived population. These papers have generally accounted for variability in conductances, with vast majority of the remaining parameters in the model held fixed. Others that have focused on conductance uncertainty include (Johnstone et al., 2016), who performed Bayesian calibration to AP data to calibrate conductances with uncertainty representing calibration error, and (Chang et al., 2015), who used a Gaussian process emulator for efficient UP and GSA after prescribing conductance uncertainty. A series of works (Sobie, 2009; Sarkar and Sobie, 2011; Sarkar et al., 2012) study the effect of variability by performing UP and GSA using multivariate regression, after introducing variability in a large number of parameters, for example up to 40 parameters including conductances, time constant scaling factors and steady-state voltage offsets in Sobie (2009), although even this was a minority of the total number of parameters in the cell model used. Hu et al. (2018) use the polynomial chaos method for efficient UP and GSA. They integrated variability in all parameters of two ionic currents (*I*_*Kto*_ and *I*_*Kur*_) of a mouse cell model with 14 currents, but uncertainty in parameters in the other 12 currents was not considered. Numerous others have integrated uncertainty in cardiac models and performed UC, UP and/or GSA, for example (Geneser et al., 2006; Sadrieh et al., 2014; Krogh-Madsen et al., 2017; Costabal et al., 2019); see also the review of Ni et al. (2018). We believe that in all cases the number of parameters varied was significantly less than the total number of parameters in the model.

It is important to appreciate that the motivation behind much of the previous work on cardiac model variability was to understand cardiac (patho-)physiology and potentially develop new therapeutic targets or methods. Our ultimate motivation is different: we wish to understand the feasibility of comprehensive UQ for cardiac-model-based tools. There are two types of model input in such tools: *fixed inputs* (quantities that take the same value every time the tool is used), and *variable inputs* (quantities that take different values when the tool is used, i.e., the inputs into the tool itself). For example, for the CiPA computational tool, which predicts pro-arrhythmic risk of a drug, all parameters within the cell model are fixed inputs, whereas drug binding parameters and drug ionic current block are variable inputs. For a hypothetical clinical tool that uses patient imaging data to generate a patient-specific heart model and provide diagnostic/therapeutic guidance to the clinician, the fixed inputs include all parameters in the underlying cell model. The imaging data are variable inputs (Recall that we are using ‘input’ as a generic term for any quantity derived from real-world data). The relatively low number of variable inputs in cardiac-model-based tools suggest UQ limited to just the variable inputs will often be feasible (see for example Chang et al., 2017). We wish to understand if it is possible to demonstrate that clinical decisions made using cardiac-model-based tools are robust to all underlying uncertainties—both in the variable and the fixed inputs—the highest possible level of rigor.

For this goal, the current state of the art for cardiac cell model UQ/GSA is far from an ideal situation in which empirically-derived uncertainty in *all* cell model parameters is known and can be propagated through the model. Here we take a concrete step toward this goal by developing a novel canine cardiac cell model containing just 36 parameters to describe six major ionic currents using Hodgkin-Huxley formulations. This cell model was developed with UQ in mind, using carefully chosen simplifications to keep the number of state variables and parameters low; the approach was motivated by the philosophy that a simple(r) model with UQ may be more useful than a complex model without UQ (National Research Council, 2012). We then perform cell model UP and GSA accounting for uncertainty in all cell model parameters (excluding three environmental parameters). For this paper input distributions will be prescribed rather than empirically-derived, a common approach in cardiac modeling (Chang et al., 2015; Hu et al., 2018) (also see Table 1 of Ni et al., 2018 for a review), here taken as an initial step. In a future paper, we plan to perform full UQ with this cell model using empirically-derived input distributions—see section Discussion. As far as we are aware, the present paper represents the first time UP and GSA has been performed accounting for variation in *all* conductance and gating kinetics parameters in a cell model. We demonstrate the general feasibility of, and elucidate some of the challenges to, the computational side to comprehensive cell model UQ. We will demonstrate that our model is robust to (sufficiently small) interacting uncertainty in all parameters—the first time this has been shown for any cardiac cell model of moderate complexity, we believe. We further study model robustness by increasing parameter uncertainty, and explore when and how new model behaviors (such as repolarization failure or loss of spike-and-dome morphology) occur, and use Monte Carlo filtering to identify which parameters are responsible. We conclude with a discussion of the implications of these results for comprehensive UQ of whole heart models, in the context of the challenges laid out above.

## 2. Methods

### 2.1. Cell Model Development: Model Structure

A novel canine cell model was developed. Transmembrane voltage was modeled using an ODE with six ionic currents

(1) $$C_{m}\frac{\text{d}V}{\text{d}t}+I_{\text{Na}}+I_{\text{K}1}+I_{\text{to}}+I_{\text{CaL}}+I_{\text{Kr}}+I_{\text{Ks}}=I_{\text{stim}}$$

where *V* is transmembrane voltage, $C_{m}=1\mu$F cm^−2^ is the specific membrane capacitance per unit area, and *I*_Na_, *I*_K1_, *I*_to_, *I*_CaL_, *I*_Kr_, and *I*_Ks_ are ionic currents (respectively: rapid sodium, inward rectifier, transient outward, L-type calcium, rapidly and slowly activating delayed rectifier). *I*_stim_ is a square wave pulse used to initialize activity. Employing a Hodgkin-Huxley formulation for each current, each current is the product of a maximal conductance, voltage-dependent gating variable and a driving force. These were modeled as:

(2) $$\begin{array}{l} I_{\text{Na}}=g_{\text{Na}}m^{3}\;h^{2}\;\left(V-E_{\text{Na}}\right) \end{array}$$

(3) $$\begin{array}{l} I_{\text{K1}}=g_{\text{K1}}\;z\;\left(V-E_{\text{K}}\right) \end{array}$$

(4) $$\begin{array}{l} I_{\text{to}}=g_{\text{to}}\;r\;s\;\left(V-E_{\text{K}}\right) \end{array}$$

(5) $$\begin{array}{l} I_{\text{CaL}}=g_{\text{CaL}}\;d\;f\;\left(V-E_{\text{Ca}}\right) \end{array}$$

(6) $$\begin{array}{l} I_{\text{Kr}}=g_{\text{Kr}}\;x_{r}\;y\;\left(V-E_{\text{K}}\right) \end{array}$$

(7) $$\begin{array}{l} I_{\text{Ks}}=g_{\text{Ks}}\;x_{s}\;\left(V-E_{\text{K}}\right) \end{array}$$

where the *g*_X_ are ion channel maximal conductances, *m*, *h*, *z*, *r*, *s*, *d*, *f*, *x*_*r*_, *y*, and *x*_*s*_ are gating variables (activation gates: *m*, *r*, *d*, *x*_*r*_, *x*_*s*_; inactivation gates: *h*, *z*, *s*, *f*, *y*), and *E*_Na_, *E*_K_, *E*_Ca_ are the Nernst potentials for sodium, potassium and calcium, respectively. Each gating variable, *Y* say, has dynamics governed by the ODE

$$\tau_{Y}\left(V\right)\frac{\text{d}Y}{\text{d}t}=Y_{\infty }\left(V\right)-Y$$

where *Y*_∞_(*V*) is the steady state activation/inactivation function and τ_*Y*_(*V*) is the voltage-dependent time constant. For steady state activation/inactivation functions, we chose sigmoid functions (ten Tusscher et al., 2004):

$$Y_{\infty }\left(V\right)={\left(1+\exp\left(\frac{\mp \left(V-E_{Y}\right)}{k_{Y}}\right)\right)}^{-1}$$

(− sign for activation gating variables, + sign for inactivation gating variables), where *E*_*Y*_ is the half-activation/inactivation voltage for that gating variable and *k*_*Y*_ > 0 controls the slope of the sigmoid. We did not model the full voltage dependence of all time constants that are typically included in other models. Instead, we chose

$$\tau_{Y}(V)=\{\begin{array}{cc} \frac{2\tau_{h0}\exp(\delta_{h}(V-E_{h})/k_{h})}{1+\exp((V-E_{h})/k_{h})}, & \text{for}\;Y=h\\ \tau_{Y}^{*}, & \text{for}\;Y=m,s,f,x_{r},x_{s}\\ 0, & \text{for}\;Y=z,r,d,y \end{array}$$

where τ_*h*0_, δ_*h*_, $\tau_{m}^{*}$, $\tau_{s}^{*}$, $\tau_{f}^{*}$, $\tau_{xr}^{*}$, $\tau_{xs}^{*}$ are positive constants. This means that gating variables *z*, *r*, *d*, *y* are assumed to instantaneously reach their steady state values. Therefore, we can replace (2) to (7) with

(8) $$\begin{array}{l} I_{\text{Na}}=g_{\text{Na}}m^{3}h^{2}\;\left(V-E_{\text{Na}}\right) \end{array}$$

(9) $$\begin{array}{l} I_{\text{K1}}=g_{\text{K1}}\;z_{\infty }\;\left(V-E_{\text{K}}\right) \end{array}$$

(10) $$\begin{array}{l} I_{\text{to}}=g_{\text{to}}\;r_{\infty }\;s\;\left(V-E_{\text{K}}\right) \end{array}$$

(11) $$\begin{array}{l} I_{\text{CaL}}=g_{\text{CaL}}\;d_{\infty }\;f\;\left(V-E_{\text{Ca}}\right) \end{array}$$

(12) $$\begin{array}{l} I_{\text{Kr}}=g_{\text{Kr}}\;x_{r}\;y_{\infty }\;\left(V-E_{\text{K}}\right) \end{array}$$

(13) $$\begin{array}{l} I_{\text{Ks}}=g_{\text{Ks}}\;x_{s}\;\left(V-E_{\text{K}}\right) \end{array}$$

Moreover, the gating variables which instantaneously reach steady state are not state variables for the system of ODEs, limiting the total number of state variables. Overall, the model has seven state variables, *V*, *m*, *h*, *s*, *f*, *x*_*r*_, *x*_*s*_, and is therefore a system of seven ODEs, and has 36 parameters—both significantly less than other cell models. The parameters are listed in Table 1.

**Table 1:** Parameters in cell model, with nominal values and derivation of value.

**Current**	**Parameter**	**Nominal value**	**Derivation**
*I*_Na_	*g*_Na_	12 mS/μF	Chosen so conduction velocity in 1D monodomain simulations was 60 cm/s (Kadish et al., 1986) – see text
	*E*_*m*_	−52.244 mV	$m_{\infty }^{3}\left(V\right)$ fit to voltage clamp data (Cordeiro et al., 2008, Figure 5D) (canine epicardial data, averaged over cells)
	*k*_*m*_	6.5472 mV	
	$\tau_{m}^{*}$	0.12 ms	Taken from Gray and Pathmanathan (2016) (based on rabbit *I*_Na_ activation data under physiological conditions, due to lack of canine *I*_Na_ activation data under physiological conditions). Together with δ_*h*_, τ_*h*0_ and some *I*_Kr_ parameters, these are the only parameters based on non-canine data.
	*E*_*h*_	−78.7 mV	Taken from Pathmanathan et al. (2015) (Table 1), which is derived from fits to canine epicardial data published in Cordeiro et al. (2008).
	*k*_*h*_	5.93 mV	
	δ_*h*_	0.799163	Taken from Gray and Pathmanathan (2016). Together with $\tau_{m}^{*}$ and some *I*_Kr_ parameters these are the only parameters based on non-canine data.
	τ_*h*0_	6.80738 mV	
*I*_K1_	*g*_K1_	0.73893 mS/μF	All derived by simultaneously fitting the *I*_K1_ model to canine epicardial voltage clamp recordings (unpublished). Experimental *E*_*K*_ was also fit as a free parameter, and *E*_*z*_ was shifted to correspond to *E*_*K*_ = −85mV
	*E*_*z*_	−91.9655 mV	
	*k*_*z*_	12.4997 mV	
*I*_to_	*g*_to_	0.1688 mS/μF	Derived by fitting the *I*_to_ model with *s* = 1 to *I*_to_ activation voltage clamp recordings [raw data behind Figure 2 of Cordeiro et al. (2012)].
	*E*_*r*_	14.3116 mV	
	*k*_*r*_	11.462 mV	
	*E*_*s*_	−47.9286 mV	Derived by fitting *s*_∞_(*V*) to *I*_to_ inactivation voltage clamp recordings [raw data behind Figure 3 of Cordeiro et al. (2012)].
	*k*_*s*_	4.9314 mV	
	$\tau_{s}^{*}$	9.90669 ms	Average of τ_*s*_(*V*) for voltages in range 10-50 mV using raw data behind Figure 2 of Cordeiro et al. (2012).
*I*_CaL_	*g*_CaL_	0.11503 mS/μF	Derived by fitting the *I*_CaL_ model to data digitized from Iyer et al. (2012) (Figure 3, epi) using values of *E*_*d*_, *k*_*d*_, *E*_*f*_, *k*_*f*_ below
	*E*_*d*_	0.7 mV	Taken from Table 2 in Iyer et al. (2012)
	*k*_*d*_	4.3 mV	
	*E*_*f*_	−15.7 mV	
	*k*_*f*_	4.6 mV	
	$\tau_{f}^{*}$	30 ms	Taken from Xiao et al. (2006) (Figure 8)
*I*_Kr_	*g*_Kr_	0.056 mS/μF	*g*_Kr_, *g*_Ks_ and $\tau_{xr}^{*}$ were jointly calibrated so simulated APD restitution matched experiment – see text
	*E*_*xr*_	−26.6 mV	Taken from Berecki et al. (2005) (Table 1), based on HEK cell data.
	*k*_*xr*_	6.5 mV	
	$\tau_{xr}^{*}$	334 ms	*g*_Kr_, *g*_Ks_ and $\tau_{xr}^{*}$ were jointly calibrated so simulated APD restitution matched experiment – see text
	*E*_*y*_	-49.6 mV	Taken from Berecki et al. (2005) (Table 1), based on HEK cell data.
	*k*_*y*_	23.5 mV	
*I*_Ks_	*g*_Ks_	0.0080 mS/μF	*g*_Kr_, *g*_Ks_ and $\tau_{xr}^{*}$ were jointly calibrated so simulated APD restitution matched experiment – see text
	*E*_*xs*_	24.6 mV	Taken from Liu and Antzelevitch (1995) (text).
	*k*_*xs*_	12.1 mV	
	$\tau_{xs}^{*}$	628 ms	
Other	*E*_Na_	65 mV	Taken from Gray and Pathmanathan (2016).
	*E*_K_	−85 mV	Based on resting membrane potential recordings in Di Diego et al. (1996)
	*E*_Ca_	50 mV	Based on data digitized from Iyer et al. (2012) (Figure 3, epi)

### 2.2. Cell Model Development: Nominal Parameter Values

‘Nominal’ parameter values were derived from either literature data or new fits to data from voltage clamp experiments on isolated canine cardiac myocytes. Table 1 provides an overview of the provenance of the parameter values. Except for three *I*_Na_ and four *I*_Kr_ parameters, all parameters were derived from canine data taken from adult canine epicardial myocytes under physiological conditions (i.e., temperature of 36-37 C, physiological extra- and intracellular ion concentrations).

Seventeen parameter values were taken directly from the literature. One (*g*_CaL_) was derived by fitting to digitized data. Eleven parameters (*E*_*m*_, *k*_*m*_; *I*_K1_ and *I*_to_ parameters) were fit to raw canine voltage clamp data. Four parameters, *g*_Na_, *g*_Kr_, $\tau_{xr}^{*}$ and *g*_Ks_, were not derived from data on their respective currents. Instead, *g*_Na_ was determined by simulating propagation in tissue (see below) to compute 1D conduction velocity, and chosen so that 1D conduction velocity was equal to 60 cm/s, to match longitudinal conduction velocity measurements in canine epicardial tissue (Kadish et al., 1986). Note that *g*_Na_ was calibrated in this way before the values of *g*_Kr_, $\tau_{xr}^{*}$ and *g*_Ks_ were finalized, but conduction velocity was found to be essentially independent of those values.

Finally, *g*_Kr_, $\tau_{xr}^{*}$, and *g*_Ks_ were simultaneously determined by fitting simulated APD95 restitution data to data taken from Volders et al. (2003) (data digitized from Figures 5A,B, average of two sets of control experiments), while constraining the ratio of peak *I*_Kr_ to peak *I*_Ks_ under 1Hz pacing matched experimental measurements (0.46 μA/cm^2^ and 0.11 μA/cm^2^; obtained by digitizing Figure 3 of Jost et al., 2013). It was determined that these three parameters are identifiable (at least locally) given this protocol. The parameters were *not* identifiable from restitution data if this constraint was not included, or if $\tau_{xs}^{*}$ was also included in the fit.

The values of the initial conditions were derived from the parameter values. Initial *V* was set to be *E*_*K*_, and initial values of the gating variables were set to be the steady state value of those gating variables at initial *V*.

### 2.3. Tissue Simulations and Numerical Solver Methods

Single cell and tissue simulations were run using Chaste, a general purpose package for computationally demanding physiological simulations. Chaste has been extensively tested and demonstrated to solve the governing equations of cardiac electrophysiology highly accurately (Niederer et al., 2011; Pathmanathan et al., 2012; Pathmanathan and Gray, 2014). For single cell simulations, the ODEs were solved in Chaste using an adaptive ODE solver. Simulation of electrical propagation through tissue was modeled using the monodomain formulation:

$$\chi \left(C_{m}\frac{\partial V}{\partial t}+I_{\text{Na}}+I_{\text{K1}}+I_{\text{to}}+I_{\text{Kr}}+I_{\text{Ks}}+I_{\text{CaL}}\right)=\nabla \cdot \left(\sigma \nabla V\right)$$

coupled to Equations (8)–(13), where χ = 1, 400cm^−1^ is the surface-area-to-volume ratio, and σ = 1.4 mS/cm is the bulk conductivity. Tissue simulations were performed using the cardiac solver in Chaste, which uses the finite element method to solve the PDE (Pathmanathan et al., 2010).

### 2.4. Uncertainty Characterization

UC involves determining probability distributions for each parameter, ideally based on experimental data and/or subject-matter expertise. The distributions should cover the parameters' uncertainty range, given the sources of uncertainty being accounted for (population variability, measurement uncertainty, etc). Determining empirally-derived probability distributions each of the parameters in Table 1 is an arguably feasible but difficult task. As discussed in section 1, here we prescribe input distributions. Specifically, we assumed that all parameters are independent and either normally-distributed (for parameters without physiological constraints) or log-normally distributed (parameters physiologically constrained to be positive). A ‘hyper-parameter’, $\hat{\sigma }$, was introduced, that controls the uncertainty across all parameters. By varying $\hat{\sigma }$, we can control the total amount of parameter uncertainty, and evaluate robustness of the model as uncertainty increases.

For parameters that are half-activation or half-inactivation voltages (*E*_*m*_, *E*_*h*_, *E*_*z*_, *E*_*r*_, *E*_*d*_, *E*_*f*_, *E*_*xr*_, *E*_*y*_, *E*_*xs*_), we chose all parameters to be normally distributed with mean equal to the nominal value (Table 1), and standard deviation proportional to (with proportionality constant $\hat{\sigma }$) a reference range of 100 mV:

(14) $$\begin{array}{l} p\;\sim \;N\left(p_{\text{nom}},\;{\left(\hat{\sigma }R\right)}^{2}\right), \end{array}$$

where *p*_nom_ is the nominal value and reference *R* = 100 mV. For conductances (*g* values), half-(in/)activation slopes (*k* values), time constants (τ values), and scaling factor δ_*h*_, all of which are necessarily positive, we set:

(15) $$\begin{array}{l} p\;\sim \;\text{lognormal }\left(\log\left(p_{\text{nom}}\right)-\frac{{\hat{\sigma }}^{2}}{2},{\hat{\sigma }}^{2}\right) \end{array}$$

Given the properties of the log normal distribution, *p* then has mean value *p*_nom_ and variance ${\hat{\sigma }}^{2}p_{\text{nom}}^{2}+O\left({\hat{\sigma }}^{4}\right)$, that is, standard deviation proportional to the nominal value, with proportionality constant, $\hat{\sigma }$.

As an illustration of the parameter ranges for a given $\hat{\sigma }$, consider parameters *g*_Na_, *E*_*m*_, *g*_CaL_, and *E*_*d*_, which are the maximal conductances and half-activation voltages of *I*_Na_ and *I*_CaL_. These have nominal values of 12 mS/μF, -52mV, 0.115 mS/μF, -0.7 mV respectively (Table 1). When $\hat{\sigma }=5\%$, the parameters become uncertain with the following 95% confidence intervals: *g*_Na_: [10.9,13.2] mS/μF, *E*_*m*_: [-61.8,-42.2] mV, *g*_CaL_: [0.104,0.127] mS/μF, *E*_*r*_: [-10.5,9.1] mV. Note that if we had chosen variances to be proportional to the absolute mean value for *all* parameters, as done elsewhere, *E*_*r*_ would be 95% certain to lie within [-0.77,-0.63] mV – much more tightly constrained than activation voltages not near zero. The use of a reference range *R* ensures inactivation/activation voltages have similar variability (for a given $\hat{\sigma }$).

Parameters *E*_K_, *E*_Na_, *E*_Ca_ were fixed as we consider them environmental parameters that can be controlled by simulating different extracellular ionic concentrations. $C_{m}$, χ and σ (monodomain conductivity) were also held fixed, for simplicity.

With these constructed distributions, we can study how the model behaves as we transition from $\hat{\sigma }=0$ (nominal model, no parameter uncertainty) to $\hat{\sigma }=1\%$ (small amount of uncertainty in all parameters (except equilibrium potentials), covering a small parameter-dependent range) to $\hat{\sigma }=5\%$ (larger uncertainty in all parameters, covering a larger parameter-dependent range). We re-iterate though that these distributions are prescribed rather than empircally-derived. Still, assessing the impact of uncertainty using prescribed distributions is common practice (Chang et al., 2015; Hu et al., 2018) and provides important insight about the behavior of computational model. Future work will focus on more accurate parameter uncertainty estimates derived from experimental data. In the discussion, section 4, we will describe how results in this paper provide information on allocating resources wisely when performing experiments for improved UC estimates.

### 2.5. Quantities of Interest

**Figure 2:**
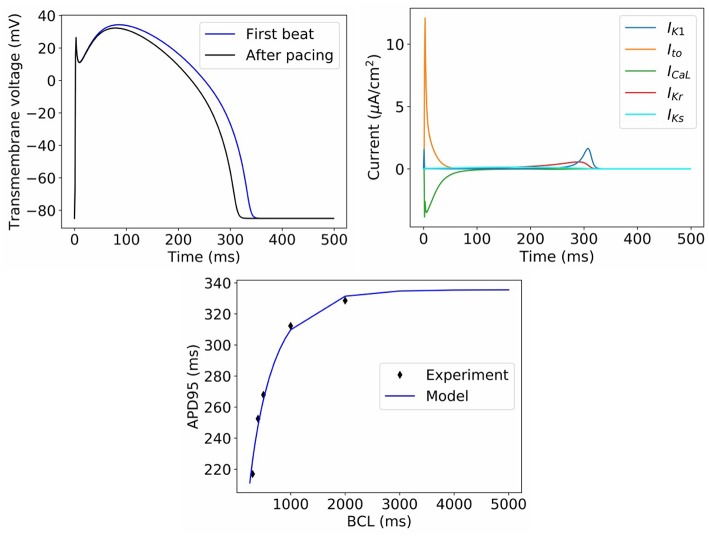
Behavior of model with nominal parameter values. **Top left**: Action potential in first beat and after pacing. **Top right**: Ionic current transients in paced action potential. **Bottom**: dynamic restitution properties, compared to experimental data used for model calibration (BCL = basic cycle length).

Various AP characteristics or quantities of interest (QOIs) were analyzed. For the upstroke, these included: **Threshold**, the minimum stimulus current needed to induce depolarization (current applied for 0.5 ms); **MaxUpstrokeVelocity**, the maximum rate of change of voltage during depolarization; **TimeOfMaxUpstrokeVelocity**, the time of this maximum; and action potential amplitude (**APA**), defined as the difference between maximum voltage during upstroke and resting membrane potential. Plateau and repolarization QOIs analyzed included: **NotchMin**, the voltage attained at the local minimum during the action potential notch (see Figure 2) (if a notch is observed), **NotchMax**, the voltage attained at the local maximum at the end of the action potential notch (if a notch is observed), and action potential duration (**APD**), the time from activation for transmembrane potential to go below -70 mV. We measured APD using a fixed threshold (-70 mV) rather than APD90 (the time for 90% repolarization from maximum voltage), because APD90 is defined using APA and APD might then be determined to be sensitive to whichever parameters strongly influence APA.

For 1D simulations using a 1 cm long strand of tissue, QOIs included the above AP characteristics at the point 0.75 cm away from the stimulus site, as well as conduction velocity (CV).

### 2.6. Global Sensitivity Analysis

Variance-based global sensitivity analysis (GSA) was performed by computing main and total Sobol sensitivity indices (Sobol', 1990). Sobol sensitivity indices provide fractional measures of the effect of the each parameter's uncertainty on the resultant variance of the model output. GSA approaches are far more computationally expensive than commonly-used *local* SA approaches, such as computing partial derivatives of the output with respect to each input, or one-at-a-time variation of each parameter. However, local SA approaches only provide information on sensitivity about the base point, resulting in incomplete or misleading information for highly nonlinear systems. GSA on the other hand explores the entire parameter space, using the distributions specified for the model inputs (section 2.4), and provides a more complete understanding of output sensitivity to inputs, including input interactions. Below is a brief introduction to this method; for a more complete introduction we refer the reader to Saltelli et al. (2008).

For a quantity of interest *q* = *q*(**p**), where *p*_*i*_ is the *i*-th parameter distributed as described in section 2.4, the expectation and variance of *q* are given by E(q)=∫q(p)dp and $\text{V}(\text{q})=\int q^{2}(\text{p})\text{d}\text{p}-{\left(\text{E}(q)\right)}^{2}$. The *i*-th *first-order Sobol sensitivity index* is defined as

$$S_{i}=V_{i}\left(q\right)/V\left(q\right)$$

where V_*i*_(*q*) = V(E(*q*|*p*_*i*_)) (inner expectation over all parameters except *p*_*i*_; outer variance over *p*_*i*_ only). The *i*-th first order Sobol index can be interpreted as the fraction of the output variance that can be attributed to the variance of parameter *p*_*i*_, not accounting for interaction with other parameters. Similarly, second-order indices measuring the fraction of the output variance that can be attributed to interaction of parameters *p*_*i*_ and *p*_*j*_ (only) are defined as *S*_*ij*_ = V(E(*q*|*p*_*i*_, *p*_*j*_)) − *S*_*i*_ − *S*_*j*_, and so on for higher-order indices. The *total sensitivity index* is given by *S*_*Ti*_ = *S*_*i*_ + Σ_*j*_*S*_*ij*_ + Σ_*j, k*_*S*_*ijk*_ + …. The total sensitivity index measures the total contribution of the parameter *p*_*i*_ to the variance of *q*, including through interactions with other parameters.

First-order and total Sobol indices were calculated using the python library SALib (Herman and Usher, 2017). The number of points sampled per dimension was chosen so that error estimates of the sensitivity indices, as provided by SALib, was small (see later figures).

For QOIs that are not computationally cheap to calculate (such as conduction velocity), computation of Sobol indices can be expensive since the total number of simulations, equal to the total number of points sampled, can be hundreds or thousands multiplied by the number of parameters. For such QOIs, the method of elementary effects, also referred to as Morris Screening (Morris, 1991), was used to identify parameters that were not influential (that is, the output has very low sensitivity to that parameter); those parameters were then excluded from the Sobol indices calculation. Morris Screening is a computationally cheap method (requiring only a few hundred simulations) that generally has a low false-positive rate when used to screen out non-influential parameters (Saltelli et al., 2008).

### 2.7. Uncertainty Propagation

Simple Monte Carlo sampling was performed using the parameter distributions [Equations (14), (15)]; histograms and statistics were computed from the resultant model outputs. Monte Carlo approaches are straightforward to implement but can be very slow to converge. In all cases the number of samples, *N*, was chosen such that the output mean (standard deviation) were converged to three (two) significant figures, when estimated using either *N* or *N*/2 samples.

### 2.8. Model Behavioral Analysis Using Monte Carlo Filtering

For some regions in parameter space, the model may transition from normal behavior to a different type of dynamics. For example, the action potential may fail to repolarize. If this occurs, probability distributions and sensitivity indices are not computable for derived quantities such as APD. There are several possibilities to consider when the model displays different classes of behavior:

1. Are the observed behaviors representative of what occurs in reality? One approach to addressing this question is to identify the mechanism underlying the behavior in the model, and confirming if that mechanism is physiologically reasonable. An alternative approach is direct statistical validation. For example, suppose a cell model fails to repolarize with small probability *p*, when parameter uncertainty representing population variability is included. It can then be asked if a random sample of isolated cardiomyocytes would also exhibit repolarization failure under identical stimuli with the same probability *p*.

If it is not believed that the observed behaviors are representative of reality, then one or both of the following should be considered:

2. Error in model form (structure of equations): one of the assumptions underlying the chosen governing equations may be being violated, at least in some regions of parameter space
3. Error in uncertainty characterization, for example some of the distributions used for input parameters may be too wide ranging and not represent reality, or there may be some correlation in reality (e.g., between *I*_Na_ half activation and inactivation Clerx, 2017) that was not properly accounted for.

Determining which path to take is difficult, and ideally requires empirically-determined input distributions, and therefore not in scope for the present paper. However, whichever of the above apply, it is very useful to identify which parameters are responsible for observed behavior. ‘Regionalized sensitivity analyses’ (Saltelli et al., 2008) are a group of methods that can be used to analyze model results exhibiting different types of behavior. They have been used in other fields but, as far as we are aware, has never been applied to cardiac models before. Here we use the Monte-Carlo filtering method (Saltelli et al., 2008), as follows: *M* points in parameter space, **p**_1_, …, **p**_*M*_, are randomly sampled, and the model solved using each. The sampled points are then split into two sets, those for which the behavior was observed, and those for which it did not. Each parameter *p*_*i*_ is then considered in turn. If *p*_*i*_ plays a role in determining whether the behavior occurs, the marginal cumulative distributions functions (CDFs) of (*p*_*i*_|Behavior occurred) and (*p*_*i*_|Behavior did not occur) will differ. The Kolmogorov-Smirnov test was be used to test whether the two CDFs were statistically different or not (α = 0.01). The Smirnov test statistic, *D*_stat_ (maximum difference between the two CDFs) was used to measure how influential a parameter was. Influential parameters were categorized as highly influential if the test statistic *D*_stat_ > 0.2.

## 3. Results

Figure 2 displays the behavior of the model using the **nominal** parameter values. Plotted is the action potential (both in the first beat following stimulation, as well as after 1 Hz pacing for 10 beats), together with the corresponding ionic current traces for the paced AP. Also plotted is APD restitution under a dynamic restitution protocol, together with the experimental data used for calibration. We do not perform validation of the cell model in this paper, since we are focused on the ability to perform UQ, which is a process that should precede validation. However, we can observe that the model reproduces canine action potential shape including spike-and-dome-morphology, and current traces take physiologically realistic shapes and magnitudes. These are emergent properties that cannot be predicted *a priori* from model equations or parameter values, that is, they are “reproduced phenomena,” using the categorization of credibility evidence discussed in Pathmanathan and Gray (2018).

**Figure 3:**
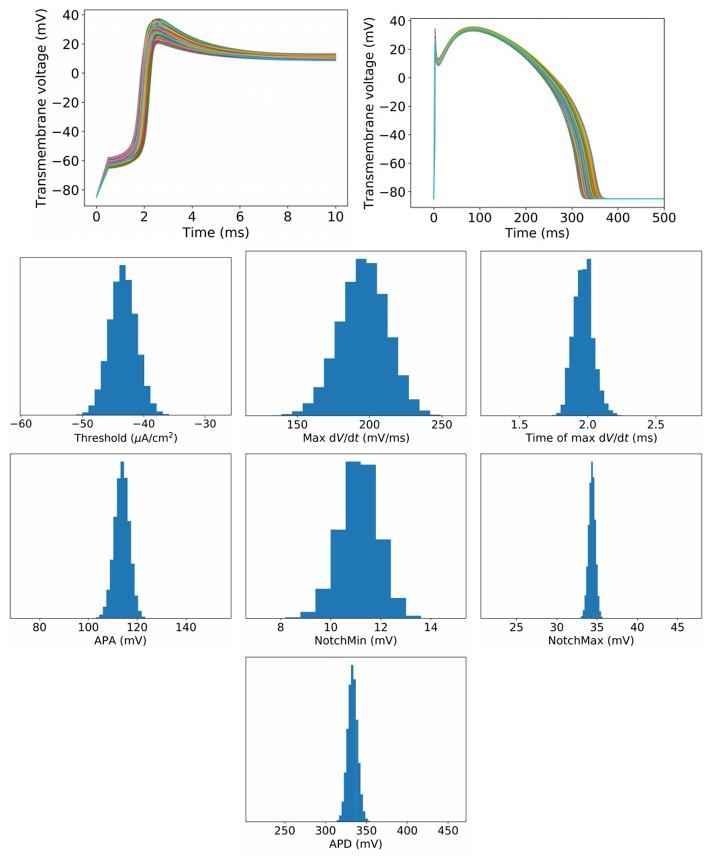
Results for $\hat{\sigma }=1\%$, which represents a small amount of uncertainty in all cell model parameters. **Top**: Upstrokes and full action potentials for 1,000 randomly sampled parameters. **Below**: converged histograms for various quantities of interest.

To begin to assess the impact of uncertainty or variability in model parameters, we first set $\hat{\sigma }=1\%$, which represents a small amount of uncertainty/variability in all parameters in the cell model. Figure 3 plots upstroke and action potential traces using 1,000 parameter sets randomly sampled using the parameter distributions Equations (14), (15) with $\hat{\sigma }=1\%$. The action potential was computed by providing a square wave stimulus with duration 0.5 ms and magnitude 1.1 times the parameter-dependent threshold stimulus. Initial conditions were those stated in section 2.2. No pacing was applied, so that these results can be fairly compared to results for greater $\hat{\sigma }$ at which pacing is not possible (see later). Also plotted in Figure 3 are converged histograms for various QOIs. The histogram *x* axes are all scaled to be approximately ±40% of the center value, which allows the width of the histograms to be fairly compared with one another.

We reiterate that these results are based on simultaneous variation of all parameters in the cell model (excluding Nernst potentials, but including *all* kinetic parameters). This is, as far as we are aware, the first time such results have been presented for a physiological cardiac cell model. Under this level of parameter uncertainty, all action potential have similar shapes compared to the nominal behavior, and no model failure or other behaviors was observed. This was not necessarily expected *a priori*: cardiac models are notoriously sensitive to small changes in parameters and it would not have been surprising if some combination of parameters, within the specified distributions, had led to anomalous behavior. Little skew is observed in the output distributions, but a range of magnitudes of variability are observed. Threshold and maximum $\frac{\text{d}V}{\text{d}t}$ have wider uncertainties than APA and APD, for example. Coefficients of variation are: Threshold: 5.0%, MaxUpstrokeVelocity: 8.4%, TimeOfMaxUpstrokeVelocity: 3.4%, APA 2.7%, APD: 1.8%.

**Figure 4:**
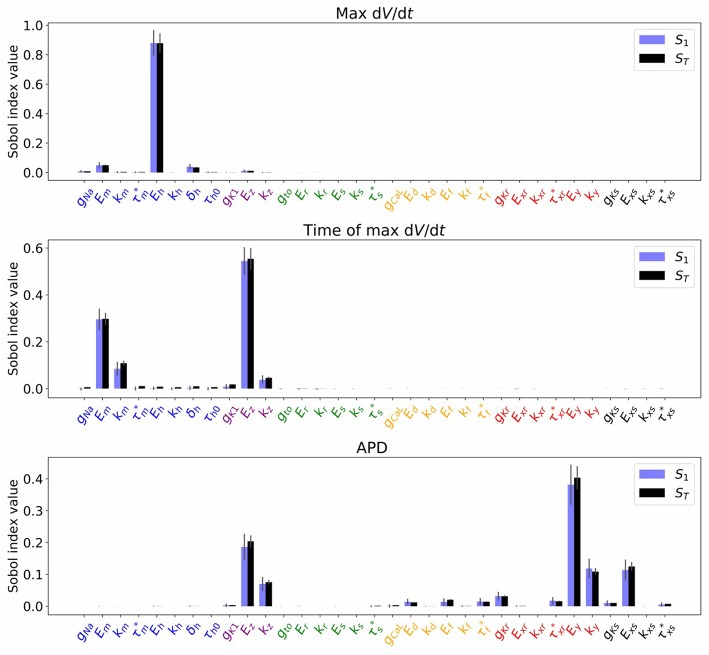
Sobol sensivitivity indices (*S*_1_: first-order; *S*_*T*_: total) for selected QOIs (MaxUpstrokeVelocity, TimeOfMaxUpstrokeVelocity, APD), with $\hat{\sigma }=1\%$, which represents a small amount of uncertainty in all cell model parameters. Solid lines are error estimates in the index.

**Figure 5:**
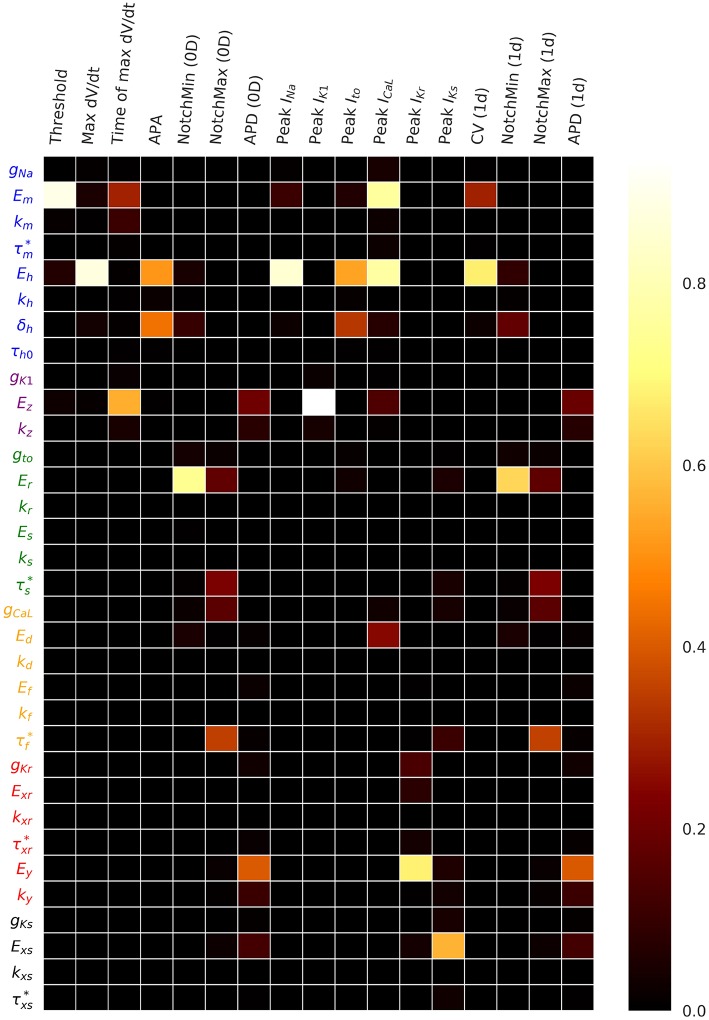
Total Sobol sensitivity indices for a range of QOIs, with $\hat{\sigma }=1\%$, which represents a small amount of uncertainty in all cell model parameters. APA is action potential amplitude. APD is action potential duration. CV is conduction velocity.

Figure 4 plots the first and total Sobol sensitivity indices for MaxUpstrokeVelocity, TimeOfMaxUpstrokeVelocity and APD. As above, we believe this is the first time sensitivities to all conductance and kinetic parameters (simultaneously) in a physiological cardiac cell model have been presented. Just one parameter, *E*_*h*_, plays a dominant role in determining MaxUpstrokeVelocity. TimeOfMaxUpstrokeVelocity is controlled by three parameters *E*_*m*_, *k*_*m*_ and *E*_*z*_, from two currents, *I*_Na_ and *I*_K1_. For APD, a variety of parameters from different currents play a role. Figure 5 displays the total Sobol indices for various QOIs, including peak currents and AP characteristics for 1D simulations of propagation. It can be seen that the sensitivity indices are very similar for APD in single cell simulations (0D) and during propagation (1D). It is important to appreciate, however, that these results are dependent on the input distributions that were prescribed in section 2.4, which were not based on experimental data, so care should be taken before drawing generalized conclusions. However, they are useful for guiding experiments and iterative model development; see discussion in section 4.

To determine if sensitivity indices or output uncertainties changed dynamically during pacing, we repeated the simulations behind Figures 3, 4 with 10 beats of 1 Hz pacing and analyzed the next AP. As expected, the mean values of the QOIs changed after pacing. However, essentially the same sensitivity indices were observed, and no appreciable changes in output uncertainty was observed (results not presented).

**Figure 6:**
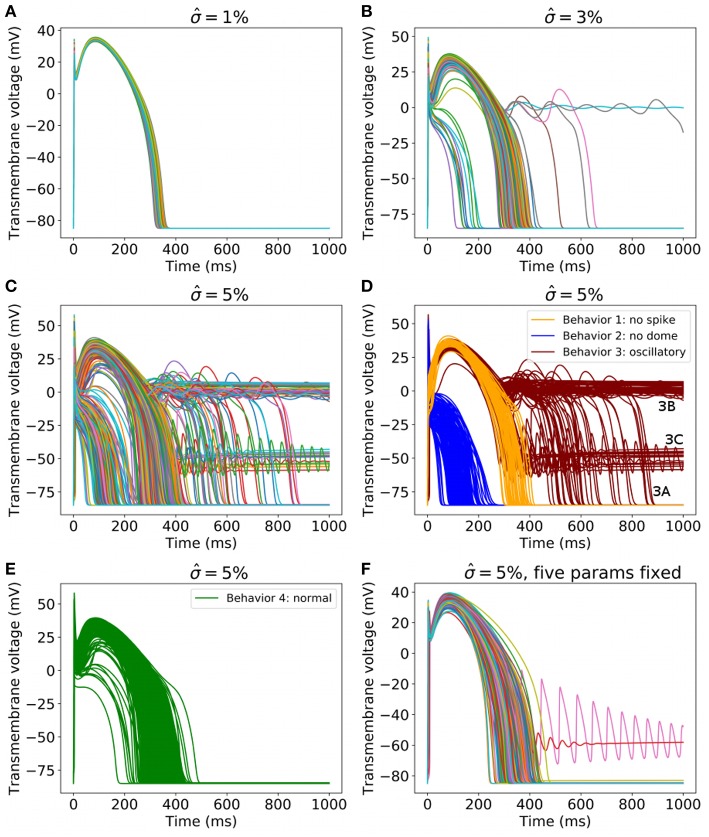
**(A–C)** 1,000 randomly sampled action potentials with: **(A)** $\hat{\sigma }=1\%$ (small amount of uncertainty in all cell model parameters); **(B)** 3% (medium uncertainty); **(C)** 5% (larger uncertainty in all cell model parameters). **(D)** Action potentials for $\hat{\sigma }=5\%$, for three classes of behavior, colored by class (blue: spike but no dome; orange: dome but no spike; red: oscillatory). **(E)** All other action potentials for $\hat{\sigma }=5\%$. **(F)** 1,000 randomly sampled action potentials with $\hat{\sigma }=5\%$ for all parameters except *E*_*h*_, δ_*h*_, *E*_*z*_, *E*_*d*_, *E*_*f*_ held fixed.

Next, we increased the uncertainty in the parameters. Figure 6 plots 1,000 randomly sampled action potentials with $\hat{\sigma }=1$, 3, and 5% (Figures 6A–C). It can be seen that for $\hat{\sigma }=3\%$, different behaviors occur; some action potentials repolarize quickly, others show early afterdepolarizations (EADs) or fail to repolarize. For $\hat{\sigma }=5\%$, a wide range of action potentials are observed. To analyze these results, we performed Monte Carlo filtering as outlined in section 2.8 to reveal which parameters are responsible for the different behaviors. To do so, we grouped the action potentials into 4 categories, as plotted in Figures 6D,E:

- **Behavior 1**: Loss of spike in spike-and-dome morphology (orange traces in Figure 6D). Defined as APs with a local maximum > 10 ms, no local minimal. Voltage continues to increase after upstroke.
- **Behavior 2**: Loss of dome in spike-and-dome morphology (blue traces in Figure 6D). Defined as APs with a local maximum < 10 ms, no local minimal. Voltage decreases monotonically after maximum upstroke voltage.
- **Behavior 3**: Oscillatory dynamics (red traces in Figure 6D). Defined as APs with more than one local minimum in action potential. These APs display oscillatory behavior such as EADs or repolarization failure. Also included in this category are any traces for which the voltage continued to rise after upstroke (as in Behavior 2) but then had two local maxima and one local minimum. These APs technically have the same number of local minima and maxima as the physiological APs but are clearly different.
- **Behavior 4**: “Normal” (green traces in Figure 6E). Defined as any AP not satisfying criteria for behaviors 1-3.

Using this categorization, the probability of different dynamics to ‘normal’ AP was: 3.2% for $\hat{\sigma }=3\%$ and 23.5% for $\hat{\sigma }=5\%$.

**Figure 7:**
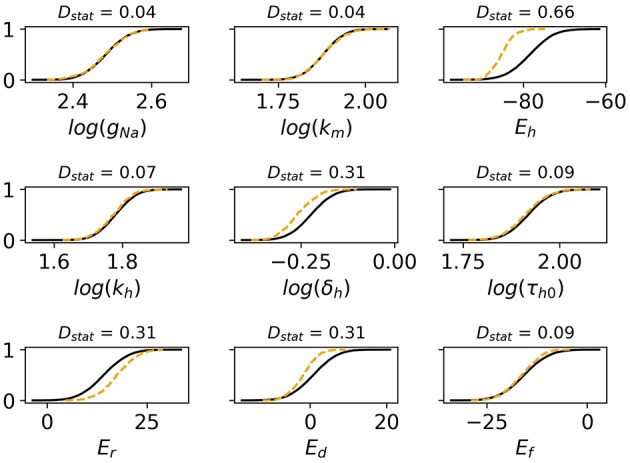
Cumulative distribution functions (CDFs) for selection of nine cell model parameters. Solid black lines are the CDFs of (*p*_*i*_|AP exhibits spike) and dashed orange lines are the CDFs of (*p*_*i*_|AP does not exhibit spike). The underlying action potentials are shown in Figures 6D,E. Non-overlapping CDFs (see e.g., *E*_*h*_, δ_*h*_, *E*_*r*_, *E*_*d*_) indicates the value of the parameter statistically impacts where AP exhibits spike or not.

Monte Carlo filtering was performed for all behaviors separately (that is, comparing Behavior 1 APs vs. all others, etc). Number of points used was *M* = 10, 000. Figure 7 plots the Monte Carlo filtering CDFs for Behavior 1, for a selection of parameters *p*_*i*_. Each panel plots the CDF of (*p*_*i*_|Behavior 1 occured) vs. the CDF of (*p*_*i*_|Behavior 1 did not occur). If the CDFs are statistically different, this indicates that *p*_*i*_ plays a role in causing Behavior 1. The two CDFs are clearly different for *E*_*h*_, δ_*h*_ *E*_*r*_, and *E*_*d*_, indicating that these parameters strongly affect whether this type of behavior occurs or not. Results of the Kolmogorov-Smirnov test are provided in Table 2. The same four parameters were found to be highly influential for Behaviors 1 and 2: *E*_*h*_, δ_*h*_ *E*_*r*_, and *E*_*d*_. Just two parameters were highly influential in determining if oscillatory behavior occurs, *E*_*d*_ and *E*_*f*_. To confirm that this analysis had accurately identified influential parameters, the $\hat{\sigma }=5\%$ simulations were repeated with *E*_*h*_, δ_*h*_ *E*_*r*_, *E*_*d*_ and *E*_*f*_ all fixed at their nominal values, but all other parameters still variable with $\hat{\sigma }=5\%$. The results are plotted in Figure 6F; it is observed that 998 out of 1,000 action potentials are now Behavior 4 (nominally normal). (The remaining small probability of other behaviors can be attributed to the “Other influential parameters” in Table 2). Note that we are **not** advocating such results be resolved by fixing influential parameters to their nominal values (instead see discussion in section 4). Simulations in Figure 6F were performed only to confirm that the Monte Carlo filtering had correctly identified influential parameters.

**Table 2:** Influential parameters for each of the three new behaviors observed in $\hat{\sigma }=5\%$ results.

**Behavior**	**Type of AP**	**Highly influential parameters (*D*_stat_ > 0.2)**	**Other influential parameters (*D*_stat_ < 0.2, *p* < 0.01)**
1 (Orange)	Dome, no spike	*E*_*h*_, δ_*h*_, *E*_*r*_, *E*_*d*_	τ_*h*0_, *E*_*f*_
2 (Blue)	Spike, no dome	*E*_*h*_, δ_*h*_, *E*_*r*_, *E*_*d*_	*k*_*h*_, τ_*h*0_, *g*_to_, $\tau_{s}^{*}$, *g*_CaL_
3 (Red)	Oscillatory	*E*_*d*_, *E*_*f*_	*E*_*m*_, *E*_*h*_, *E*_*z*_, *k*_*z*_, *g*_CaL_, *k*_*f*_, *g*_Kr_, *E*_*y*_

*Colors correspond to Figure 6D*.

As discussed in section 2.8, one should consider if the different behaviors observed are representative of reality or not. We cannot do the statistical validation outlined in section 2.8 since the input uncertainty was prescribed rather than empirically-derived, but we can consider if the behaviors observed and the influential parameters behind them are consistent with known physiological understanding. Behaviors 1 and 2, loss of spike or loss of dome from spike-and-dome morphology, would be expected to be related to changes in *I*_to_ and *I*_CaL_, and indeed *E*_*r*_ (*I*_to_ half-activation voltage) and *E*_*d*_ (*I*_CaL_ half-activation voltage) are highly influential in these behaviors. Also highly influential are *I*_Na_ inactivation parameters *E*_*h*_ and δ_*h*_; it is not clear why this occurs and suggests that further investigation is warranted into these aspects of the model equations and/or on the magnitude of prescribed uncertainty in these parameters. Behavior 3, oscillatory dynamics, is expected to be caused by *I*_CaL_ window currents, and therefore by *E*_*d*_ and *E*_*f*_, the half-activation and half-inactivation voltages for *I*_CaL_. This is consistent with the highly influential parameters identified for this set of action potentials. We can identify three distinct classes of sub-behavior in the oscillatory results (see Figure 6D):

- **Behavior 3A**: APs with early afterdepolarizations (*V* at resting potential at *t* = 1, 000 ms);
- **Behavior 3B**: APs exhibiting repolarization failure (*V* > −25 mV at *t* = 1, 000 ms); and
- **Behavior 3C**: APs exhibiting low voltage oscillations (−75 mV < *V* < −25mV at *t* = 1, 000 ms).

Behavior 3C appears unphysiological and may be genuine model failure. To analyze these results further, we repeated the Monte Carlo filtering analysis for each of the three sub-classes; results are presented in Table 3. The low voltage oscillatory APs are seen to be caused by *E*_*m*_, *E*_*h*_, and *E*_*z*_. Further analysis is required to determine if this is due to a problem with the model equations or due to the prescribed uncertainty in *E*_*m*_, *E*_*h*_, *E*_*z*_ being unrealistically large.

**Table 3:** Influential parameters for each of the three sub-behaviors observed in $\hat{\sigma }=5\%$ oscillatory action potentials.

**Behavior**	**Type of AP**	**Highly influential parameters (*D*_stat_ > 0.2)**	**Other influential parameters (*D*_stat_ < 0.2, *p* < 0.01)**
3A	Early afterdepolarizations	*E*_*d*_, *E*_*f*_	*E*_*z*_, *k*_*z*_
3B	Repolarization failure	*E*_*d*_, *E*_*f*_	*k*_*z*_, *k*_*f*_, $\tau_{f}^{*}$, *g*_Kr_, *E*_*y*_, *k*_*y*_
3C	Low voltage oscillations	*E*_*m*_, *E*_*h*_, *E*_*z*_	*k*_*z*_

**Figure 8:**
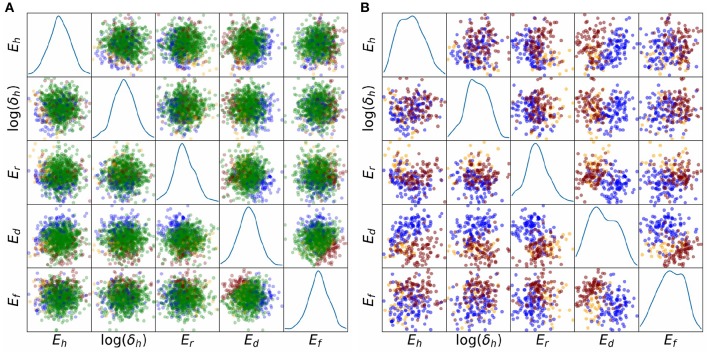
Scatter plots of parameter values, colored by behavioral class, for $\hat{\sigma }=5\%$ results, representing relatively large uncertainty in all parameters. Five important parameters are plotted, *E*_*h*_, δ_*h*_, *E*_*r*_, *E*_*d*_, *E*_*f*_. These were identified using behavioral analysis (see text). Blue points: points in parameter space for which APs display spike but no dome; orange points: APs display dome but no spike; red points: APs are oscillatory; green points: APs are normal. **(A)** All points. **(B)** Points corresponding to non-normal APs only. The underlying APs are shown in Figures 6D,E.

Finally, we investigated whether potential correlations between parameters could be responsible for some of the observed behaviors. Figure 8 plots the sampled points in parameter space, colored by behavior class. The plot is limited to the five-dimensional space of (*E*_*h*_, log(δ_*h*_), *E*_*r*_, *E*_*d*_, *E*_*r*_), which were the parameters identified to be highly influential in determining whether any of the four main behaviors occurs or not (Table 2). Figure 8A plots all points including those corresponding to normal APs (green), Figure 8B plots the points corresponding to Behaviors 1–3 only. Visual inspection suggests a possible negative correlation between *E*_*h*_ and log(δ_*h*_), since the (*E*_*h*_, log(δ_*h*_)) subplot in Figure 8A shows few normal AP points where *E*_*h*_ and log(δ_*h*_) are *both* small. Visual inspection of Figure 8A also suggests a possible positive correlation of *E*_*d*_ and *E*_*f*_ for points corresponding to normal APs. Moreover, the (*E*_*d*_, *E*_*f*_) figure in Figure 8B suggests that oscillatory behavior (red points) is associated with *E*_*f*_ − *E*_*d*_ taking relatively large values, and no-dome behavior (blue points) is associated with *E*_*f*_ − *E*_*d*_ taking relatively small values. To quantify the analysis, we computed correlation coefficients for all pairs of (*E*_*h*_, log(δ_*h*_), *E*_*r*_, *E*_*d*_, *E*_*r*_), for the points in parameter space which corresponded to normal APs. They were: -0.12 for (*E*_*h*_, log(δ_*h*_)), 0.13 for (*E*_*r*_, *E*_*d*_), 0.15 for (*E*_*f*_, *E*_*d*_); all other correlation coefficients were <0.05. These results could provide direction in future voltage clamp experiments which simultaneously estimate multiple physiological properties in the same cell.

## 4. Discussion

This paper was motivated by the fact that UQ and SA are understood to be important for biomedical computational models—as demonstrated by their inclusion in the ASME V&V40 Standard (ASME V&V 40, 2018)—yet a fully comprehensive UQ/SA analysis for whole heart models is impossible with current technology (as discussed in section 1). We have presented a novel cardiac cell model which has relatively few (36) parameters, which enabled us to perform UQ and GSA accounting for uncertainty in all conductance and kinetic parameters, albeit with prescribed input distributions. Future work will focus on empirically-derived input distributions. We also analyzed the robustness of the model by increasing the underlying uncertainty to study the different behaviors that arise, and determined which parameters were responsible for those behaviors. Since our overarching motivation is to explore the feasibility of cardiac model UQ, we structure the first part of the discussion around the four main challenges to performing cardiac UQ that were described in section 1.2. We then discuss the implications of this work for clinically-relevant applications of cardiac models.

### 4.1. Challenge 1: Large Number of Inputs (Including Model Parameters) in Cardiac Models

We described in section 1.2 how the sheer number of quantities in cardiac models that are derived from physiological data is one of the greatest difficulties for cardiac UQ. Especially problematic are the number of parameters in modern cell models, for example, the OR11 model (O'Hara et al., 2011) has more than 250 numeric quantities in its governing equations that were derived from data in some way. Therefore, we developed a novel 36 parameter model, where each parameter has a clear physiological interpretation, for which it is arguably feasible to introduce empirically-derived uncertainty in all parameters. The model has various simplifications such as instantaneous gating and no intracellular ionic concentrations, which means it is not suitable for applications involving the mechanisms of excitation-contraction coupling, ischemia or reproducing the exact details of ionic current traces during voltage clamp experiments. However, it is suitable for investigating the impact of simultaneously variation in all parameters, and could serve as a starting point for development of incrementally more complex models, for example for developing a ‘minimally-complex’ model for a chosen application, for which comprehensive UQ remains possible. In general, there is a trade-off between less complex models which will likely exhibit less physiologically accurate behavior vs. more complex models which are less transparent and have greater numbers of uncertain inputs (Huberts et al., 2018). For cardiac models the crucial question is: *can simpler models be as predictive as complex models for clinically-relevant applications?* If so, simpler models have the advantage that fully testing robustness to parameter uncertainty is possible. This question has not been explored in any great depth but deserves greater attention—most recent research has been focused on whether the currently-available complex models are predictive for clinical applications.

Even with simple(r) models, characterizing the true uncertainty (whether population variability or measurement uncertainty) in all the parameters is a difficult experimental task (even ignoring the correlation question, discussed below). Sensitivity analysis and regionalized sensitivity analysis following UC with prescribed input distributions, as performed here, can provide guidance for allocating resources wisely. For example, the results in Table 2 and Figure 5 suggest it *may* be an inefficient use of resources to run experiments to estimate the population variability in *g*_Na_, $\tau_{m}^{*}$, *g*_K1_, *g*_to_, *k*_*r*_, *E*_*s*_, *k*_*s*_, *k*_*d*_, *k*_*f*_, *k*_*xr*_, $\tau_{xr}^{*}$, *g*_Ks_, *k*_*xs*_, or $\tau_{xr}^{*}$, since none of these parameters appear in Table 2 or influence any of the QOIs in Figure 5. However, once improved uncertainty estimates have been obtained for the other parameters, the sensitivity analysis should be repeated, using crude but wide-ranging uncertainty in the above parameters, to confirm that they are still uninfluential. Also, and very importantly, when testing a model for a specific model application, the sensitivity analysis should be performed *for the QOI to be used in decision-making*, not just generic QOIs. Accordingly, the above results on parameter sensitivity may not extrapolate to spiral wave dynamics.

There are several methods that can be used to characterize the uncertainty due to population variability in cell model parameters, and the choice depends on the various factors, including the type of parameter, method used to derive the nominal parameter value (Table 1) and the availability of data from individual cells. For parameters for which voltage clamp data is available from individual cells, probability distributions can be derived by fitting parameters to individualized data, and then fitting probability distributions to the resultant parameters, similar to what has already been done for *E*_*h*_ and *k*_*h*_ in Pathmanathan et al. (2015). This is not possible for parameters for which only averaged data is available; for those a reasonable first approximation may be to use similar magnitude uncertainty as for corresponding parameters in other currents. Parameters that are calibrated using the full AP model, as opposed to derived from voltage clamp data or the literature (four parameters—see Table 1) may need to be re-calibrated with uncertainty, perhaps using Bayesian methods Johnstone et al., 2016). When suitable data is not available, a common approach is uncertainty elicitation using subject matter expertise (Morris et al., 2014). Obtaining/estimating uncertainty in time constant parameters will likely pose the greatest challenge, for two reasons. First, experimentally measuring time constants is more difficult than steady-state behavior. Second, time constants have known voltage-dependence, but in our model we approximated them as being independent of voltage (except for τ_*h*_). This raises the question of what exactly is meant by population variability in this quantity. One solution is to define the parameter more precisely, for example as the average time constant over a pre-specified voltage range. This is a then well-defined quantity for which it is meaningful to ask what the population variability is.

### 4.2. Challenge 2: Difficulty in Measuring Correlation Between Model Inputs

Many of the parameters in cardiac cell models might be correlated across the species population. For example, Clerx describes how half-activation and half-inactivation voltages for *I*_Na_ appear to be correlated in human (Milstein et al., 2012; Clerx, 2017) shows potential correlation between *g*_Na_ and *g*_K1_. The second major challenge mentioned in section 1 is the fact that identifying such correlations is experimentally very difficult, if not impossible. Again, model results can be used to guide experiments. First, correlations involving uninfluential parameters (see previous subsection) do not need to be considered. For influential parameters, distributions of model outputs assuming different input correlation structures could be computed, to determine parameters for which introducing correlation makes a difference. Alternatively, parameters which give rise to different types of model behavior could be assessed (as was presented in Figure 8), which may provide insight into which parameters to investigate experimentally. For example, we could hypothesize from the results in Figure 8 that the half-activation (*E*_*d*_) and half-inactivation (*E*_*f*_) voltages for *I*_CaL_ are positively correlated in reality (for canine), since larger values of |*E*_*d*_ − *E*_*f*_| were associated with different types of non-physiological action potentials. This hypothesis is based on assumptions about the accuracy of the model form, and therefore needs to be verified experimentally.

### 4.3. Challenge 3: Computational Cost of Running Large Numbers of Whole Heart Simulations

We did not run any 2D or 3D simulations for this paper, nor was computational cost a focus of this paper. In fact, two of the methods used, Sobol sensitivity analysis and simple Monte Carlo sampling, are fairly computationally demanding even for single-cell analyses (although initial Morris Screening (section 2.6) reduces the computational cost for computing the sensitivity indices). Therefore, our results provide limited insight into the challenge of running large numbers of whole heart simulations to perform UQ for whole-heart simulation-based outputs. We expect that emulators of whole-heart models will need to be developed to overcome this challenge; see ongoing research (Chang et al., 2015; Ghosh et al., 2018; Lawson et al., 2018). Note that sensitivity indices were very similar for QOIs in 0D and 1D (Figure 5), as were output distributions (results not presented), suggesting that uninfluential parameters in single-cell simulations may be uninfluential in tissue simulations. It is not clear that this conclusion will hold for simulations involving arrhythmias however.

### 4.4. Challenge 4: Unclear Path Forward If Model Failure Occurs

Even if comprehensive UC and UP could be performed with cardiac models, it is likely that a range of behaviors will be observed, and many common model outputs will not be computable for many of the sampled points in parameter space, for example APD when repolarization failure occurs. If this occurs, scientific conclusions or clinical decisions based on that model output are not robust to the input uncertainty. The final challenge described in section 1 was the fact that in situations such as this, it may not be clear how to proceed. In this paper we have observed how a range of AP dynamics occur as uncertainty in parameters is increased, and demonstrated how Monte Carlo filtering can be used to identify exactly which parameters are influential for each behavior (see section 3). This provides a pathway to investigate the root cause for the observed behaviors. In section 2.8 we discussed how one possibility is that the model is accurately reproducing different dynamics that occurs in reality. If this is not the case, the conclusion should be that the model failure occurred, in which case there are two options. Either (i) the model is an inaccurate representation of reality, at least in some regions of parameter space; and/or (ii) the uncertainty representations of some of the parameters are inappropriate in some way (e.g., do not represent true variability or do not account for correlations that occur in reality). For example, the results from section 3 suggest experimentally investigating whether *I*_CaL_ half-activation and half-inactivation voltages are correlated, because larger values of |*E*_*f*_ − *E*_*d*_| were associated with both oscillatory APs and with loss of dome. Section 3 also showed how uncertainty in *I*_Na_ half-activation and half-inactivation voltages *E*_*m*_ and *E*_*h*_, and in *I*_K1_ half-inactivation voltages *E*_*z*_, was responsible for non-physiological low voltage oscillatory behavior, suggesting focused investigation into those aspects of the model equations or refinement of uncertainty ranges for those parameters.

### 4.5. Significance

We end with a discussion of implications of our work for clinical applications of cardiac models. We focus on cardiac models as predictive tools, such as the *in silico* model used in the CiPA program for assessing drug cardiotoxicity (Li et al., 2018; Strauss et al., 2018). As discussed in section 1.3, for such tools, inputs can be categorized as either fixed (taking the same value every time the tool is used) or variable (the converse). For example, for the CiPA computational tool, all parameters within the cell model are fixed inputs, whereas drug binding parameters and drug ionic current block are variable inputs. It is undeniably important to perform UQ in *variable inputs*. Typically, uncertainty in those values with be due to measurement uncertainty, and if the tool output is not robust to this measurement uncertainty then the tool is not reliable. The importance of performing UQ in the fixed inputs is more debatable. For fixed inputs, the greatest source of uncertainty will often be due to population variability. Here, UQ in the fixed inputs can provide confidence that clinical decisions derived from the tool are robust to that underlying variability, especially as clinical trial results are extrapolated to a broader patient population. As an illustrative example, consider a hypothetical tool that has two inputs, patient-specific height (variable input) and average patient weight (fixed input). The clinical decision made using the tool certainly needs to be robust to any measurement error in patient height. Since any given patient may not be average weight, UQ to demonstrate that the clinical decision is robust to the uncertainty in weight, for the intended patient population, would provide additional confidence in the tool, and potentially reduce the need for a clinical trial cohort to fully cover the range of weights in the intended patient population. This is a simplistic example but the same ideas apply for variable and fixed inputs in patient-specific clinical tools based on personalized cardiac models (Gray and Pathmanathan, 2018).

The results in this paper represent a step toward cardiac model-based tools that are demonstrably robust to underlying uncertainties, for both fixed and variable inputs, but there remain many challenges to be overcome. For one, we used prescribed input uncertainty in this paper, the next step is to investigate AP variability with empirically-derived input distributions. If (when) overly large AP variability is observed, this will need to be addressed by one or more of: (i) refining the model governing equations; (ii) refining parameter uncertainty estimates; (iii) determining which parameters are correlated; and/or (iv) identifying parameters that should potentially be personalized in clinical applications because their uncertainty due to population variability leads to too much output uncertainty. This will likely result in several iterations of the following: model form refinement; parameter uncertainty characterization; robustness analysis as performed here; and analysis of different behaviors observed (section 2.8). This will need to be performed initially for simple pacing protocols (as performed here) and then arrhythmia-inducing protocols. Our model is canine, but the same approach can used to develop a human model, although parameterization will be more challenging. There are various open questions about how best to integrate uncertainty into tissue simulations (Ni et al., 2018). Finally, simplified models will be need to be shown to be predictive for clinical applications, and incrementally improved if there are not. This could be performed iteratively with UQ in each step as additional complexity is added. An incremental bottom-up approach may also allow formal methods for accounting for *model form uncertainty* (structural uncertainty) to be used (Mirams et al., 2016). Overall, although there is a long way to go, we believe this approach—development and iterative refinement of simplified models for which UQ in all parameters is possible—represents a complementary pathway for developing models for clinical cardiac applications, in comparison to the traditional approach where established high complexity models are used in clinical applications and UQ in all parameters is not possible. We expect that such research will reveal more information about the consequences of physiological variability in cardiac models, and on the general credibility of cardiac (and other physiological) models.

## Ethics Statement

This investigation conforms to the Guide for Care and Use of Laboratory Animals published by the National Institutes of Health (The Eighth Edition of the Guide for the Care and Use of Laboratory Animals [NRC 2011]). All protocols were approved by the Institutional Animal Care and Use Committee.

## Author Contributions

PP devised the project, determined and implemented analytic methods, run simulations and analysis, and wrote paper. JC provided raw data from canine voltage clamp experiments. RG developed cell model and provided feedback on paper.

### Conflict of Interest Statement

The authors declare that the research was conducted in the absence of any commercial or financial relationships that could be construed as a potential conflict of interest.

## Footnotes

^1^https://clinicaltrials.gov/ct2/show/NCT03536052

^2^https://commonfund.nih.gov/sites/default/files/RM16-008SPARC4DRCOT3.pdf

^3^For example, consider the simple model *y*(*x*_1_, *x*_2_) = *x*_1_ + 10*x*_2_. LSA would conclude that *y* is 10 times more sensitive to *x*_2_ than *x*_1_. GSA, however, requires information on the distributions of the inputs. Suppose *x*_1_ ~ *N*(0, 1) and *x*_2_ ~ *N*(0, 0.01). Then uncertainty propagation would reveal that *y* ~ *N*(0, 1.1), and GSA would reveal that with 91% of *y*'s variance can be attributed to the uncertainty in *x*_1_ and 9% to the uncertainty in *x*_2_.
